# Energy Utilization in Premature Neonates Undergoing Screening for Retinopathy of Prematurity

**DOI:** 10.3390/pediatric17020029

**Published:** 2025-03-03

**Authors:** Alena M. Pentecost, Danilo S. Boskovic, Alexis Antimo, Udochukwu Oyoyo, Christopher C. Perry, Jennifer Dunbar, Andrew Hopper, Danilyn M. Angeles

**Affiliations:** 1 Department of Basic Sciences, School of Medicine, Loma Linda University, Loma Linda, CA 92350, USA; 2 Division of Biochemistry, Department of Basic Sciences, School of Medicine, Loma Linda University, Loma Linda, CA 92350, USA; dboskovic@llu.edu (D.S.B.);; 3 Department of Earth and Biological Sciences, School of Medicine, Loma Linda University, Loma Linda, CA 92350, USA; 4 Department of Dental Education Services, School of Dentistry, Loma Linda University, Loma Linda, CA 92350, USA; 5 Department of Ophthalmology, School of Medicine, Loma Linda University, Loma Linda, CA 92350, USA; 6 Department of Pediatrics, School of Medicine, Loma Linda University, Loma Linda, CA 92350, USA

**Keywords:** retinopathy of prematurity screening, energy utilization, uric acid, ATP degradation, oxygen desaturation, oxygen support, premature neonate, high-performance liquid chromatography

## Abstract

Background/Objectives: Premature neonates are at risk for retinopathy of prematurity (ROP) and routinely undergo screening exams that involve substantial physical manipulation, often causing significant signs of pain, despite pain-relieving interventions. It remains unclear whether these exams affect energy utilization, cellular hypoxia, and clinically significant events, and whether receiving supplemental oxygen affects these relationships. This work examines the effects of ROP screening on (1) urinary uric acid-to-creatinine concentration ratios ([UA]/[Cr]), a known marker of ATP degradation, hypoxia, and oxidative stress; and (2) clinically significant events (apnea, bradycardia, gastric residuals, and oxygen desaturations) in premature neonates on room air or oxygen support. Methods: This prospective pilot study included premature neonates requiring ROP screening examinations at Loma Linda University’s NICU. Urinary [UA]/[Cr], measured by high-performance liquid chromatography, and clinical events, documented by prospective medical chart review, were analyzed pre- and post-exam in subjects on room air (*n* = 18) or on oxygen support (*n* = 20). Statistical analyses included a generalized linear mixed model for urinary [UA]/[Cr] and Wilcoxon signed rank tests for clinical events. Results: A significant time effect (*p* = 0.010) was observed for urinary [UA]/[Cr], with higher levels at 0–12 (*p* = 0.023) and 12–24 (*p* = 0.023) hours post-exam. Subjects receiving oxygen support had more total (*p* = 0.028) and more severe (*p* = 0.026) oxygen desaturations. Conclusions: ROP examinations may increase energy utilization in premature neonates, with those receiving oxygen support being particularly susceptible to oxygen desaturations post-exam. Further research is needed to clarify the full impact of the procedure and to identify strategies to minimize stress associated with these screening examinations.

## 1. Introduction

Premature neonates are at risk for retinopathy of prematurity (ROP), a disorder in the development of the retinal vasculature [1]. Peripheral retinal cryotherapy or laser photocoagulation are known to be effective in treating ROP and reducing unfavorable structural and visual outcomes, such as blindness [1,2,3,4,5]. However, these procedures are only effective if performed in a timely fashion, which requires that premature neonates at risk for ROP are screened using carefully timed retinal screening examinations, sometimes at repeated intervals [1]. Unfortunately, these examinations, which include the use of mydriatic eye drops and physical manipulation of the eye via lid speculum and scleral indentation, are documented to increase signs of pain, despite the use of pain-relieving interventions [6,7,8], and have been linked to the transient arrest of gastrointestinal peristalsis, as well as to necrotizing enterocolitis [9].

**Figure 1:**
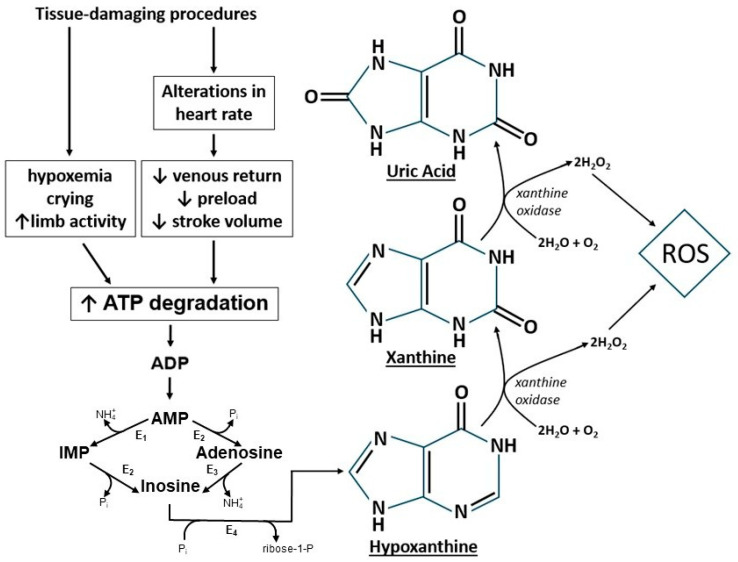
Effect of tissue-damaging procedures on breakdown of ATP to uric acid (adapted from prior reports [12,13]). E1, AMP deaminase; E2, 5′ nucleotidase; E3, adenosine deaminase; E4, purine nucleoside phosphorylase.

We previously reported that during ROP screening, despite stable peripheral oxygen saturation (SpO_2_), the mesenteric regional tissue oxygen saturation (mStO_2_) significantly decreased from baseline in all subjects, with greater reductions in mStO_2_ in neonates receiving oxygen support compared to those breathing room air [10]. Our lab also demonstrated that tissue-damaging procedures can increase concentrations of plasma uric acid, a breakdown product of ATP degradation, in both rabbits [11] and human premature neonates [12] (see mechanistic schematic in Figure 1, adapted from our prior work [12,13]). Similarly, the urinary uric acid-to-creatinine concentration ratio ([UA]/[Cr]) is a known biomarker of ATP degradation, as well as hypoxia or oxidative stress [13,14,15,16]. However, it remains unclear whether ROP screening, a procedure known to increase signs of pain and stress, affects energy utilization, cellular hypoxia, and clinical events in these neonates, and whether receiving supplemental oxygen affects these relationships.

The primary objective of this prospective pilot study is to compare acute changes in urinary [UA]/[Cr] before and after ROP screening in two cohorts of premature neonates: those breathing room air and those receiving supplemental oxygen support. We also studied the effects of ROP examination on clinical events, including apnea, bradycardia, gastric residuals, and oxygen desaturations, as indicators of the neonate’s clinical status, before and 24 h after ROP examination. We hypothesized that ROP examination increases urinary [UA]/[Cr] and adverse clinical events, particularly in neonates receiving oxygen support.

## 2. Materials and Methods

### 2.1. Subject Enrollment

The outcomes reported here were derived from the same cohort of subjects studied previously, using methods described earlier [10]. Briefly, this prospective pilot study was conducted at Loma Linda University Children’s Hospital Neonatal Intensive Care Unit (NICU). Recruitment occurred between March 2015 and September 2017. Premature neonates less than 37 weeks of estimated gestational age were eligible if they met the following inclusion criteria: (a) required ROP eye examination, (b) met the American Academy of Pediatrics and the American Academy Ophthalmology joint statement’s screening guidelines [1], and (c) parents gave consent in writing. Infants were excluded from the study if they met any of the following criteria: (a) scheduled for laser eye surgery on the day of planned examination, (b) with intraventricular hemorrhage greater than grade 3, diagnosed by head ultrasound according to Papile classification [17], (c) receiving any of the following medications: morphine, fentanyl, methadone, midazolam, lorazepam, muscle relaxants, phenobarbital, phenytoin, and levetiracetam, (d) with renal injury (plasma creatinine > 1.5 mg/dL), (e) with severe cyanotic congenital heart disease (receiving inotropes and/or infusion of prostaglandin E_2_), (f) with severe respiratory distress (unstable arterial blood gases), or (g) with gastrointestinal dysfunction (displaying symptoms of necrotizing enterocolitis).

### 2.2. ROP Screening Procedure and Documentation of Clinical Data

**Figure 2:**
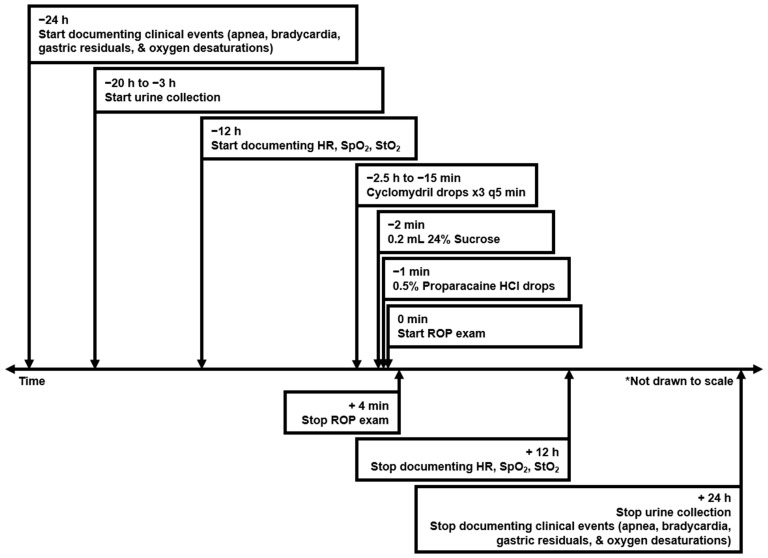
Study timeline (adapted from [10]). Heart rate (HR), systemic peripheral oxygen saturation (SpO_2_), and regional tissue oxygen saturation (StO_2_) outcomes were previously reported [10].

Figure 2 illustrates the general study timeline based on the standardized protocol, adapted from our previously reported work with additional details [10]. Before the ROP exam, subjects’ pupils were dilated using Cyclomydril, a combination of 0.2% cyclopentolate hydrochloride (to inhibit pupil constriction) and 1% phenylephrine hydrochloride (to dilate the pupil). To minimize procedural pain, 0.2 mL of Sweet-Ease^TM^, a 24% sucrose solution was administered orally, to the buccal mucosa. Oral sucrose was shown to reduce signs of pain in neonates, likely through the activation of endogenous opioid pathways [18]. The practice of giving sucrose to neonates prior to painful procedures is the current standard of care in many NICUs across the globe, including our own. Topical anesthetic eye drops, consisting of 0.5% proparacaine hydrochloride, were administered within minutes prior to the approximately four-minute ROP exam. Each eye was examined individually using a binocular indirect ophthalmoscope. A speculum held the eyelids open, while a depressor was used to manipulate the globe’s position as needed by the ophthalmologist.

Despite efforts to standardize the screening procedure, some deviations occurred. The timing of the administration of sucrose, proparacaine hydrochloride, and the ROP exam varied somewhat, according to clinical need, but most procedures occurred within minutes of the standardized protocol timeline. Of the 38 subjects, 35 received oral sucrose before the ROP examination. One subject did not receive sucrose, another received sucrose 54 min before the examination, and one received sucrose three times prior to the examination. One neonate received Cyclomydril twice prior to examination, one underwent an ROP exam lasting significantly longer than four minutes, and one received a blood transfusion during the 12–24 h study period.

For 24 h before and after the exam, medical records were prospectively reviewed for clinically significant apnea, bradycardia, oxygen desaturation events requiring clinical interventions (stimulation, oxygen supplementation), as well as the volume of gastric residuals. Apnea is defined as cessation of respiration lasting at least 20 s [19]. Bradycardia is characterized by a heart rate below 80 beats per minute [20]. Oxygen desaturation is defined as SpO_2_ below 90%, categorized as mild (85–89% SpO_2_), moderate (81–84% SpO_2_), or severe (≤80% SpO_2_) [21]. The ROP diagnosis and vision status at hospital discharge were retrospectively obtained from the medical records.

### 2.3. Measurement of Urinary Uric Acid-to-Creatinine Concentration Ratios

Urine samples were collected and analyzed using previously described methods [22]. A cotton ball was placed over the urethral meatus and subsequently removed with each diaper change, approximately every 3–4 h. Samples uncontaminated by stool were stored at 4 °C before processing to minimize any potential chemical reactions that could alter our findings until samples could be analyzed. Urine was obtained from each cotton ball using pressure via syringe, then centrifuged at 4 °C and 20,000× *g* for 10 min, filtered with a Millex syringe filter (Low Protein Binding Durapore PVD filter, 0.45 μm, 13 mm; Millipore Corp, Billerica, MA, USA), and stored at −80 °C for subsequent analysis. Prior validation data from our lab, including published [22] and previously unpublished data (Supplemental Table S1), demonstrated that purines and creatinine remained stable under our sampling, storing, and processing conditions.

Baseline urine samples from pre-ROP exam diaper changes were pooled into one sample for processing and analysis, while post-exam samples were stored separately for each diaper change, processed and analyzed as individual samples, and subsequently aggregated, as described below. In some cases, stool contamination and/or clinical events disrupted urine collection, such that some individual samples were not available for a given neonate. Baseline samples were collected 9 to 20 h before the examination, depending on when parental consent was obtained. However, for one subject, baseline urine collection began just over three hours prior to the examination. Additionally, differences in care schedules meant that four neonates received care every four hours versus every three hours, and one neonate was transitioned from a three-hour to a four-hour care schedule. These samples were assigned to the appropriate time windows based on their documented collection times, similarly to neonates on a 3 h care schedule.

The 38 subjects provided 260 samples, which were analyzed via high-performance liquid chromatography (HPLC) to yield urinary [UA]/[Cr] determinations. Uric acid and creatinine levels were measured as previously published by our laboratory [22], with an adaptation of the HPLC method described by George et al. [23]. Urine samples were thawed, sonicated, then transferred in 200 µL aliquots to a microcentrifuge tube containing 1 × 10^−7^ mol (13.51 µg) of 2-aminopurine (2-Ap) (formula weight = 135.13 g/mol) as the internal standard. 2-Aminopurine was chosen as the standard for HPLC because it has purine characteristics, as well as distinct absorbance and elution profiles from the purines of interest [24]. The samples were then analyzed, with three separate injections per sample, using HPLC (Waters 996 PDA, 600 controller, and 717plus autosampler; Millipore Corp) by injecting 35 μL onto a Supelcosil LC-18-S 15 cm × 4.6 mm, 5 μm column (SGE; Austin, TX, USA), with isocratic conditions including a mobile phase of 10 mM potassium dihydrogen phosphate buffer at a pH of 4.7 and a flow rate of 1.0 mL/min. Uric acid, creatinine, and 2-aminopurine were quantified by obtaining peak areas at the appropriate retention times and wavelengths (288 nm, 235 nm, 305 nm, respectively) [22]. The area ratios of each compound to 2-Ap were converted to concentrations with the use of standard curves (catalog numbers: UA—#U0881, Cr—#C4255, 2-Ap—#A3509; Sigma Aldrich, St. Louis, MO, USA). To eliminate variabilities in urine output, uric acid concentration was normalized to creatinine concentration. Creatinine synthesis has been reviewed and described in detail elsewhere [25].

Of the 260 analyzed samples, 18 were excluded due to issues such as collection errors, coelution, unidentifiable purines, instrumentation errors, or lack of reliable triplicate measurements, and 11 were excluded due to coefficients of variation over 10%. Fifty samples had UA/2-Ap areas exceeding the standard curve’s maximum, nine exceeded the Cr/2-Ap maximum, and 37 exceeded both. As re-analysis with wider standard curve ranges was not feasible, these samples were included to evaluate temporal trends rather than precise quantities, recognizing potential underestimation of the true effects. After data clean-up, 90% of sample measurements were retained. Contributions per subject ranged from two datapoints to nine datapoints. To address variabilities in clinical courses and sample timing, post-exam samples were grouped into 0–12 and 12–24 h windows, by averaging all samples within each window, and a one-hour overlap in sample timing was permitted, allowing collections up to 13 h post-exam for the 12 h window. The final cleaned and aggregated dataset contained 108 datapoints from 38 subjects: 6 subjects (16%) contributed two datapoints, and 32 subjects (84%) contributed three datapoints.

### 2.4. Statistical Analysis

This study exhibits features typical of a pilot study, and no formal power calculation was conducted beforehand. Instead, the sample size was determined based on prior related studies [10,13]. Patient characteristics for the two groups (room air versus oxygen support) were summarized using descriptive statistics, with mean ± standard deviation for continuous variables and counts with percentages for categorical variables. Statistical significance for demographic variables was determined using Mann–Whitney *U* or Pearson’s Chi-Square (*Χ*^2^) tests, with 2-tailed, exact *p*-values reported for each.

The primary outcome, urinary [UA]/[Cr] in response to the ROP exam, involved repeated measures, with datapoints at 12 h intervals, as described above. Assumptions of normality and equal variance were assessed. Due to the non-normally distributed nature of the data, a generalized linear mixed model was applied to accommodate the data structure, with gamma distribution and log link function. The primary analysis included fixed effects for time (baseline and each subsequent 12 h increment), group (room air versus oxygen support), and their interaction, while incorporating subject ID as a random effect with repeated measures. Post hoc pairwise contrasts for the significant fixed effects were performed, with the Bonferroni method applied to adjust for potential Type I error from multiple comparisons. Due to the small sample size and the associated limitations in controlling for potential confounders, statistically significant demographic variables were examined for their relationship with urinary [UA]/[Cr] at each aggregated time window. Spearman’s rho (*ρ*) tests were used for continuous variables, and a Pearson’s *Χ*^2^ test with two-tailed exact *p*-values was applied for the categorical variable of race/ethnicity.

Clinical events, including episodes of apnea, bradycardia, gastric residuals, and oxygen desaturations, were analyzed pre- and post-exam using Wilcoxon signed rank tests, with two-tailed exact *p*-values reported. Statistical significance was defined as *p* < 0.05.

## 3. Results

### 3.1. Subject Demographics

The original study enrolled 42 subjects [10]. However, urinary [UA]/[Cr] measurements were available for only 38 subjects. The demographic characteristics of these 38 subjects are summarized in Table 1. As expected, the two groups showed significant differences at birth in weight (*U* = 74.50, *p* = 0.002) and estimated gestational age (EGA; *U* = 75.50, *p* = 0.002), and at the time of the exam in weight (*U* = 79.00, *p* = 0.003), corrected gestational age (CGA; *U* = 89.50, *p* = 0.007), oxygen delivery mode (*U* = 38.00, *p* < 0.001), and maximum FiO_2_ before (*U* = 54.00, *p* < 0.001) and after (*U* = 45.00, *p* < 0.001) the exam. Race/ethnicity also differed significantly (*p* = 0.049) between groups. However, no significant differences were observed in 1 min or 5 min APGAR scores, illness severity defined by the Score for Neonatal Acute Physiology with Perinatal Extension II (SNAPPE-II) [26,27], or gender.

### 3.2. Urinary Uric Acid-to-Creatinine Concentration Ratios Before and After ROP Exam

**Figure 3:**
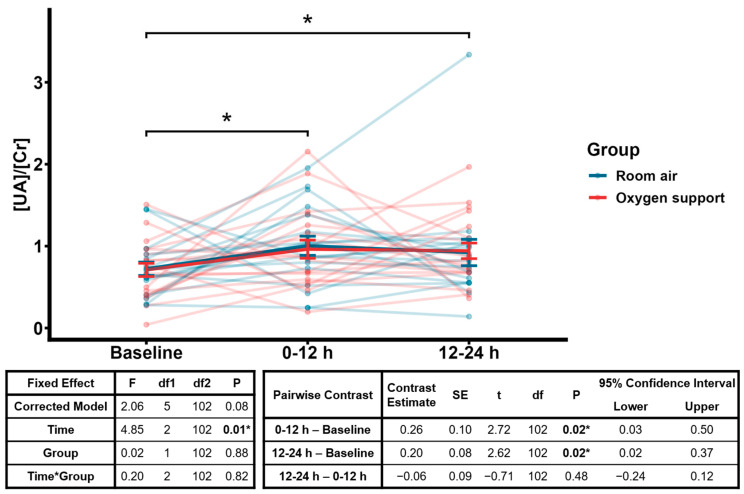
Urinary [UA]/[Cr] at baseline before ROP exam and 0–12 and 12–24 h after ROP exam. Each thin line represents an individual subject (*n* = 38), while thick lines indicate group means. Error bars represent standard error of the mean. Left table shows results of generalized linear mixed model with gamma distribution and log link, with fixed effects of time, group, and time-by-group interaction effect, and a random effect of subject ID. Right table shows results of post hoc Bonferroni-adjusted pairwise contrasts for the time effect; significant contrasts are identified in the figure with bars and asterisks. * *p* ≤ 0.05.

When modeling the effects of the ROP exam on urinary [UA]/[Cr], we found a statistically significant time effect (*F* = 4.85, *p* = 0.01; Figure 3). Post hoc comparisons revealed significant differences at both 0–12 h (*t* = 2.72, *p* = 0.02) and 12–24 h (*t* = 2.62, *p* = 0.02) compared to baseline, with no significant differences between 0–12 and 12–24 h. However, there were no significant effects for group, the time-by-group interaction effect, or the corrected model. Supplemental Table S2 shows the concentration of uric acid, together with the concentration of creatinine, as well as the ratio of uric acid-to-creatinine, for each oxygen status group across timepoints.

When assessing for potential confounding effects of demographic variables on urinary [UA]/[Cr] over time, no significant associations were observed between demographic variables that were significantly different between the two groups and urinary [UA]/[Cr] at baseline, 0–12, or 12–24 h (Supplemental Table S3). Subgroup analyses revealed no statistically significant correlations in the room air group. However, in the oxygen support group, birthweight was positively correlated with urinary [UA]/[Cr] at 0–12 h post-exam (*ρ* = 0.64, *p* = 0.003).

### 3.3. Clinically Significant Events Before and After ROP Examination

**Figure 4:**
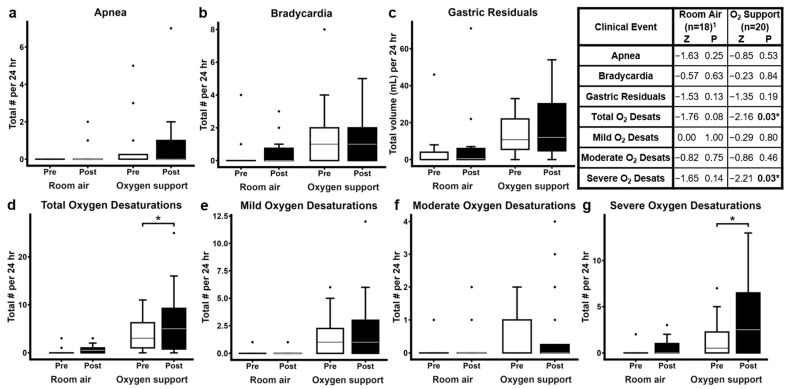
Clinical events documented by prospective medical chart review in subjects in room air or receiving oxygen support 24 h before and after ROP exam. (**a**) Total number of apnea events, (**b**) total number of bradycardia events, (**c**) total volume of gastric residuals, (**d**) total number of total oxygen desaturations, (**e**) total number of mild oxygen desaturations, (**f**) total number of moderate oxygen desaturations, (**g**) total number of severe oxygen desaturations. Figures display box plots, with box indicating median and IQR, upper whisker extending to the largest observation less than or equal to 1.5 × IQR, lower whisker extending to the smallest observation greater than or equal to 1.5 × IQR, and outliers outside of 1.5 × IQR. Table shows results of Wilcoxon signed rank tests with 2-tailed exact *p*-values. * *p* ≤ 0.05. ^1^ Sample size was *n* = 18 for all variables in the room air group, except for gastric residuals, for which *n* = 16. O_2_—oxygen; Desats—desaturations.

Analysis of clinical events post-ROP exam (Figure 4) revealed no significant differences in clinically significant apnea events, bradycardia events, or gastric residual volumes in either the room air or oxygen support groups. However, the oxygen support group experienced a significant increase in total oxygen desaturation events (*Z* = −2.162, *p* = 0.03; Figure 4d), while the room air group showed an increase that approached, but did not reach, statistical significance (*Z* = −1.76, *p* = 0.08; Figure 4d). When clinically significant oxygen desaturations were classified by severity, the oxygen support group showed a significant increase post-ROP exam in severe desaturations (*Z* = −2.21, *p* = 0.03; Figure 4g), but not in mild or moderate desaturations, while the room air group did not experience significant changes in oxygen desaturations of any particular severity.

### 3.4. ROP Diagnosis at Discharge

Subjects’ ROP diagnoses at hospital discharge are presented in Table 2. There were no significant differences in ROP stage or zone at discharge. One subject in the oxygen support group required laser photocoagulation therapy. The last recorded vision assessments indicated that ROP had resolved in all subjects.

## 4. Discussion

### 4.1. Relationship Between ROP Examination and Urinary Uric Acid-to-Creatinine Concentration Ratios

Although baseline urinary [UA]/[Cr] ratios did not differ between subjects in room air or on oxygen support, we found that these ratios were significantly higher after the ROP exam in all subjects, at both 0–12 and 12–24 h post-exam (Figure 3). These findings suggest that the ROP exam procedure may have altered ATP metabolism in this cohort. Interestingly, we observed no significant correlations between demographic characteristics and urinary [UA]/[Cr] at any timepoint when all subjects are considered (Supplemental Table S3), suggesting that the significant time effect observed in the generalized linear mixed model (Figure 3) is not driven by differences in demographics. However, given the variability in individual responses, the observed effect may reflect an overall trend, rather than a predictable effect of examination in individual subjects. While the physiological basis for the positive correlation between birthweight and urinary [UA]/[Cr] at 0–12 h post-exam in oxygen support subjects (Supplemental Table S3) remains unclear, the significantly lower birthweight in this group may have attenuated the expected increase in urinary [UA]/[Cr] post-exam.

### 4.2. ROP Examination and Clinically Significant Oxygen Desaturations

We observed significantly higher numbers of total and severe oxygen desaturation events in the oxygen support group post-exam, along with a non-significant trend toward increased total oxygen desaturations in the room air group (Figure 4). This trend may reach significance with a larger sample size. However, our prior work also demonstrated significantly lower mesenteric oxygen saturations during ROP examination in oxygen support subjects [10]. In the current study, no significant differences in apnea events were found pre- and post-exam in either group. However, others reported increased apnea events 24–48 h after eye examination, with significant differences in oxygen desaturation events between neonates with or without apnea [28]. Notably, the use of Cyclomydril may also contribute to increases in oxygen desaturation events [29]. Oxygen desaturation metrics have been reported to contribute to the prediction of increased clinical risk for ROP, as well as other adverse outcomes such as bronchopulmonary dysplasia, severe intraventricular hemorrhage, and prolonged neonatal intensive care unit stay [30]. Thus, by increasing oxygen desaturations, the ROP exam may itself inadvertently increase risk of ROP.

### 4.3. Conceptualizing the Relationships Between ROP Examination, Increased Urinary Uric Acid-to-Creatinine Concentration Ratios, and Increased Oxygen Desaturations

The current study provides additional support of the significant link between painful procedures, such as ROP examinations, and ATP degradation as well as hypoxia. We previously demonstrated that plasma uric acid concentration increased in animals after central catheter placement [11] and in premature human neonates after tape removal [12], suggesting that tissue-damaging procedures increase uric acid levels. We also previously observed that premature neonates who did not receive either oral glucose or oral sucrose analgesia before painful procedures had significantly higher urinary [UA]/[Cr] levels compared to those who received a sweet solution [13], further supporting the link between pain and increased urinary [UA]/[Cr].

Although nearly all subjects in this study received oral sucrose before the ROP exam, urinary [UA]/[Cr] levels still increased after the exam. Despite the brief duration of the ROP examination and the use of pain-relieving interventions, this increased [UA]/[Cr] appears to imply that the screening for ROP is particularly stressful and tissue-damaging, including the significant physical manipulation such as eyelid speculum insertion, scleral indentation, and eye globe manipulation, all of which can cause discomfort and pain [6,7,8,10,31]. Both during and after this process, neonates experience increased motor activity (crying, increased limb movement) [32], as well as heightened metabolic demands from a two-fold increase in sympathetic nervous system activity [8]. Our findings support the understanding that increased energy demands lead to higher cellular ATP utilization and its subsequent breakdown into uric acid (Figure 1). This breakdown of ATP then necessitates increased ATP synthesis [33], which may subsequently increase oxygen consumption, as oxygen is required to generate the proton motive force necessary for ADP phosphorylation to ATP in the mitochondrial electron transport chain [33,34]. This increase in oxygen consumption could present as clinical oxygen desaturations, and the newly synthesized ATP could also be degraded to contribute to additional uric acid production. Lower oxygen saturations could result in reduced ATP synthesis [35], which may then require the breakdown of existing ATP stores to continue to meet energy demands, potentially resulting in further production of uric acid. Uric acid would then be released from the cytosol into the extracellular space, enter the circulation, and be filtered by the kidneys for excretion in urine [36,37,38].

It is also possible that there is an enhancing effect on ATP utilization by the sucrose given prior to the exam, specifically the fructose subunit. Fructose metabolism bypasses the regulated phosphofructokinase step in the liver, consequently increasing the energy demands [39,40,41]. Alternatively, the increased urinary [UA]/[Cr] may highlight the inconclusive effectiveness of oral sucrose in alleviating the pain and discomfort associated with the ROP examination procedure [7,42,43,44].

### 4.4. Clinical Implications

These findings suggest that ROP screening effectively identifies neonates at risk for blindness and facilitates early treatment. However, the care of premature neonates during ROP screening exams needs a revised strategy to minimize associated pain. Studies recommend both non-pharmacological interventions (such as oral sucrose, human milk, facilitated tucking, swaddling, and non-nutritive sucking) and pharmacological options (including topical anesthesia, paracetamol, fentanyl, and dexmedetomidine) [31,45]. Slevin et al. found that premature infants placed in a nest-like position with soft boundaries during ROP exams exhibited less discomfort, as measured by movement and crying [46]. However, in a separate study, swaddling, holding, and administering a 24% sucrose solution from 15 min before to 15 min after the eye examination did not significantly affect vital signs (heart rate, respiratory rate, and oxygen saturation) compared to a control group [47].

Short NIDCAP (Newborn Individualized Developmental Care and Assessment Program) interventions positively affect pain and stress responses during routine procedures like diaper changes and weighing [48,49]. Kleberg found that NIDCAP interventions—such as modifying the infant’s environment, adjusting positioning, pacing the procedure, using a gentle approach, providing non-nutritive sucking, and stabilizing the head—were effective in reducing both stress and discomfort during clinical eye examinations [50]. Given the lack of universally accepted pain relief standards for ROP screening and established guidelines for post-examination care and feeding, our findings highlight the urgent need for well-designed, randomized controlled trials with adequately large sample sizes. These trials should evaluate the most effective pharmacological and non-pharmacological pain management strategies during and after ROP screening exams, while minimizing adverse effects, such as increased energy expenditure, respiratory depression, and feeding disturbances.

### 4.5. Limitations

Our study is primarily limited by its small sample size. While we observed interesting trends in the data, some did not reach significance. These limitations could be addressed by conducting a larger, well-powered study. Additionally, the analysis of urine samples in this cohort proved challenging, as neonates do not urinate on a fixed schedule, and many samples need to be excluded due to stool contamination, resulting in missing datapoints that complicate statistical interpretation. The integration of purines is often challenging due to poor peak resolution. However, our manual integration methods were designed to minimize noise as much as possible. The presence of samples with UA/2-Ap and Cr/2-Ap area ratios outside of the maximum range of the respective standard curves likely underestimated the true differences in urinary [UA]/[Cr] over time. Finally, urine sample collection and medical record documentation of apnea, bradycardia, gastric residuals, and oxygen desaturation events involved a large team of clinical staff, which introduced variability. Providing additional training for nurses participating in the study could help reduce this limitation [10]. Despite these challenges, this pilot study offers valuable insights into the relationship between ROP examinations, ATP degradation, and hypoxia, as reflected in neonatal urine samples and clinical records.

## 5. Conclusions

In summary, this pilot study identifies a potential mechanism connecting retinopathy of prematurity screening examinations to increased urinary uric acid-to-creatinine concentration ratios in premature neonates, irrespective of their oxygen support status. Although only neonates receiving oxygen support experienced significantly more oxygen desaturations after the examination, a larger sample size might have detected a similar effect in neonates breathing room air. Given the vulnerabilities of this population and the likelihood that these neonates will experience multiple screening exams, it is crucial to establish improved examination protocols that minimize distress, pain, and hypoxia. Future research should focus on further elucidating this mechanism with larger, sufficiently powered sample sizes and refined protocols, as well as conducting randomized clinical trials designed to test both pharmacological and non-pharmacological methods of pain relief during examinations. For neonates requiring these invasive procedures, this work is critical to minimize the negative consequences of retinopathy of prematurity screenings and improve both short- and long-term health outcomes in this vulnerable group.

## Figures and Tables

**Table 1:** Subject demographics. Continuous variables presented as mean ± SD; Categorical variables presented as count (%); ^1^ Mann–Whitney U test; ^2^ Pearson’s Χ^2^ test; *p*-values are 2-tailed and exact; * *p* ≤ 0.05. EGA—Estimated gestational age; APGAR—Appearance, Pulse, Grimace, Activity, and Respiration; SNAPPE-II—Score for Neonatal Acute Physiology with Perinatal Extension-II; CGA—corrected gestational age; CPAP—continuous positive airway pressure; HFNC—high-flow nasal cannula; NAVA—neurally adjusted ventilatory assist; NC—nasal cannula; NIMV—non-invasive mechanical ventilation.

	Room Air (*n* = 18)	Oxygen Support (*n* = 20)	Test Statistic ^1^	*p* ^1^
**Birthweight (g)**	1232 ± 438	859 ± 255	74.50	0.002 *
**EGA at Birth (wk)**	29.4 ± 2.2	27.0 ± 2.2	75.50	0.002 *
**APGAR, 1 min**	5.1 ± 1.9	3.9 ± 2.6	131.00	0.15
**APGAR, 5 min**	7.0 ± 1.2	6.5 ± 2.2	177.50	0.95
**SNAPPE-II**	15.7 ± 11.0	22.8 ± 14.5	139.50	0.23
**Gender**	Female: 8 (44%) Male: 10 (56%)	Female: 8 (40%) Male: 12 (60%)	0.08 ^2^ (df = 1)	1.00 ^2^
**Race/Ethnicity**	Asian/Hispanic: 2 (11%) Black/Hispanic: 1 (6%) Black/Not Hispanic: 4 (22%) Other/Hispanic: 0 (0%) Other White/Hispanic: 1 (6%) Other White/Not Hispanic: 1 (6%) White/Hispanic: 4 (22%) White/Not Hispanic: 3 (17%) >1 Race/Hispanic: 2 (11%)	Asian/Hispanic: 1 (5%) Black/Hispanic: 0 (0%) Black/Not Hispanic: 0 (0%) Other/Hispanic: 2 (10%) Other White/Hispanic: 2 (10%) Other White/Not Hispanic: 1 (5%) White/Hispanic: 12 (60%) White/Not Hispanic: 2 (10%) >1 Race/Hispanic: 0 (%)	13.80 ^2^ (df = 8)	0.049 * **^2^**
**Weight at Exam (g)**	2388 ± 729	1721 ± 553	79.00	0.003 *
**CGA at Exam (wk)**	36.4 ± 2.7	34.1 ± 2.3	89.50	0.007 *
**O_2_ Delivery Mode** **at Exam**	Room air	CPAP: 5 (25%) HFNC: 10 (50%) NAVA: 1 (5%) NC: 2 (10%) NIMV: 2 (10%)	38.00 ^2^ (df = 5)	<0.001 *^2^
**Max FiO_2_ 24 h** **Pre-exam (%)**	21.0 ± 0.0	29.5 ± 8.0	54.00	<0.001 *
**Max FiO_2_ 24 h** **Post-exam (%)**	21.0 ± 0.0	33.4 ± 11.9	45.00	<0.001 *

**Table 2:** Discharge demographics. Data are presented as count (%). Pearson’s *Χ*^2^ test; *p*-values are 2-sided. * Subject was treated with laser photocoagulation therapy. NS = no statistic is calculated because the variable is a constant.

	Room Air (*n* = 18)	Oxygen Support (*n* = 20)	*Χ* ^2^	*p*
**ROP Location ** **at Discharge**	Zone 1: 0 (0%) Zone 2: 9 (50%) Zone 3: 7 (39%) Mature: 2 (11%)	Zone 1: 0 (0%) Zone 2: 12 (60%) Zone 3: 6 (30%) Mature: 2 (10%)	0.401 (df = 2)	0.818
**ROP Severity** **at Discharge**	Mature: 2 (11%) Stage 0: 13 (72%) Stage 1: 2 (11%) Stage 2: 1 (6%) Stage 3: 0 (0%)	Mature: 2 (10%) Stage 0: 9 (45%) Stage 1: 2 (10%) Stage 2: 6 (30%) Stage 3: 1 * (5%)	5.208 (df = 4)	0.267
**Last Documented** **ROP Diagnosis**	ROP resolved: 18 (100%)	ROP resolved: 20 (100%)	NS	NS

## Data Availability

The datasets generated and/or analyzed during the current study are available from the corresponding author (D.M.A.) on reasonable request.

## Supplementary Materials

The following supporting information can be downloaded: https://www.mdpi.com/article/10.3390/pediatric17020029/s1. Table S1: Concentrations of uric acid and creatinine, together with uric acid-to-creatinine concentration ratios, in urine analyzed under various processing conditions; Table S2: Concentrations of uric acid and creatinine, and the concentration ratio of uric acid-to-creatinine at baseline, 0–12 h, and 12–24 h by oxygen support status; Table S3: Association of demographic variables with urinary [UA]/[Cr] at baseline, 0–12 h, and 12–24 h by oxygen support status.

## Abbreviations

The following abbreviations are used in this manuscript:

2-Ap	2-Aminopurine
CGA	Corrected gestational age
EGA	Estimated gestational age
HPLC	High-performance liquid chromatography
NICU	Neonatal intensive care unit
NIDCAP	Newborn Individualized Developmental Care and Assessment Program
ROP	Retinopathy of prematurity
SNAPPE-II	Score for Neonatal Acute Physiology with Perinatal Extension II
SpO_2_	Systemic peripheral oxygen saturation
mStO_2_	Mesenteric regional tissue oxygen saturation
[UA]/[Cr]	Uric acid-to-creatinine ratio
