# Supplementary material for: Energy Utilization in Premature Neonates Undergoing Screening for Retinopathy of Prematurity
Source: Pediatr Rep. 2025 Mar 3;17(2):29. doi: 10.3390/pediatric17020029 (PMC11932259; doi:10.3390/pediatric17020029)
Supplement: Supplementary file 1 [file pediatrrep-17-00029-s001.zip › pediatrrep-3445547-supplementary.pdf]

Supplemental Information

Energy Utilization in Premature Neonates Undergoing Screening for Retinopathy of Prematurity

Alena Pentecost[1], Danilo S. Boskovic[2,3], Alexis Antimo[1], Udochukwu Oyoyo[4], Christopher C. Perry[2], Jennifer Dunbar[5], Andrew Hopper[6], Danilyn M. Angeles[1,6*]

[1]Department of Basic Sciences, School of Medicine, Loma Linda University, Loma Linda, CA 92350, USA
[2]Division of Biochemistry, Department of Basic Sciences, School of Medicine, Loma Linda University, Loma Linda, CA 92350, USA
[3]Department of Earth and Biological Sciences, School of Medicine, Loma Linda University, Loma Linda, CA 92350, USA
[4]Department of Dental Education Services, School of Dentistry, Loma Linda University, Loma Linda, CA 92350, USA
[5]Department of Ophthalmology, School of Medicine, Loma Linda University, Loma Linda, CA 92350, USA
[6]Department of Pediatrics, School of Medicine, Loma Linda University, Loma Linda, CA 92350, USA
[*]Corresponding author. Email: dangeles@llu.edu

Processing Condition	[UA]	[Cr]	[UA]/[Cr]
**Straight**	1385	3190	0.4342
**Cotton**	1328	3165	0.4195
**Cotton 3 h**	1228	3168	0.3876
**Mean**	1313	3174	0.4138
**SD**	79.66	13.69	0.0238
**SE**	45.99	7.902	0.0138
**CV**	6.065	0.4312	5.758

Supplemental Table S1. Concentrations of uric acid and creatinine, together with uric acid-to-creatinine concentration ratios, in urine analyzed under various processing conditions. This validation was carried out by comparing uric acid and creatinine levels measured from a) Straight: urine without contact with cotton, b) Cotton: urine from urine-soaked cotton balls, and c) Cotton 3 h: urine from urine-soaked cotton balls incubated for 3 hours at room temperature. SD = Standard Deviation, SE = Standard Error, CV = Coefficient of Variation (calculated as Mean/SD*100).

	[UA]			[Cr]			[UA]/[Cr]		
	Baseline	0-12 h	12-24 h	Baseline	0-12 h	12-24 h	Baseline	0-12 h	12-24 h
**All Subjects (N=38)**	1275 (55, 3202)	1226 (242, 3763)	1094 (384, 2541)	1958 (456, 5405)	1604 (112, 5243)	1714 (376, 4687)	0.664 (0.041, 1.507)	0.877 (0.197, 2.154)	0.826 (0.140, 3.338)
**Room Air (n=18)**	1294 (317, 2511)	1153 (330, 2182)	1104 (384, 2541)	1988 (697, 3849)	1550 (791, 5243)	1731 (499, 4128)	0.678 (0.281, 1.453)	0.952 (0.247, 1.953)	0.818 (0.140, 3.338)
**Oxygen Support (n=20)**	1254 (55, 3202)	1407 (242, 3763)	1085 (435, 2444)	1958 (456, 5405)	1769 (112, 4312)	1698 (376, 4687)	0.664 (0.041, 1.507)	0.873 (0.197, 2.154)	0.834 (0.363, 1.967)

Supplemental Table S2. Concentrations of uric acid and creatinine, and the concentration ratio of uric acid-to-creatinine at baseline, 0-12 hours, and 12-24 hours by oxygen support status. Data are presented as median (minimum, maximum) with concentrations given in μM.

			Baseline	0-12 h	12-24 h
All Subjects	Birthweight (g)	ρ	0.01	0.07	-0.13
		P	0.94	0.70	0.44
		n	35	37	36
		df	33	35	34
	EGA at Birth (wk)	ρ	-0.09	-0.03	-0.18
		P	0.63	0.85	0.30
		n	35	37	36
		df	33	35	34
	Weight at Exam (g)	ρ	0.07	0.28	-0.08
		P	0.70	0.10	0.66
		n	35	37	36
		df	33	35	34
	CGA at Exam (wk)	ρ	-0.01	0.15	-0.05
		P	0.94	0.38	0.77
		n	35	37	36
		df	33	35	34
	Race/Ethnicity	X^2	280.00	291.07	288.00
		P	0.21	0.31	1.00
		n	35	37	36
		df	264	280	280
Room Air	Birthweight (g)	ρ	-0.07	-0.43	-0.32
		P	0.80	0.08	0.22
		n	16	18	17
		df	14	16	15
	EGA at Birth (wk)	ρ	-0.25	-0.41	-0.10
		P	0.36	0.09	0.70
		n	16	18	17
		df	14	16	15
	Weight at Exam (g)	ρ	-0.13	0.18	-0.20
		P	0.64	0.48	0.44
		n	16	18	17
		df	14	16	15
	CGA at Exam (wk)	ρ	-0.21	0.15	-0.06
		P	0.43	0.55	0.81
		n	16	18	17
		df	14	16	15
	Race/Ethnicity	X^2	112.00	126.00	119.00
		P	1.00	1.00	1.00
		n	16	18	17
		df	105	119	112
Oxygen Support	Birthweight (g)	ρ	0.10	0.64	0.12
		P	0.68	**0.003***	0.63
		n	19	19	19
		df	17	17	17
	EGA at Birth (wk)	ρ	-0.05	0.35	-0.16
		P	0.83	0.14	0.51
		n	19	19	19
		df	17	17	17
	Weight at Exam (g)	ρ	0.26	0.42	0.10
		P	0.29	0.07	0.69
		n	19	19	19
		df	17	17	17
	CGA at Exam (wk)	ρ	0.16	0.15	0.09
		P	0.52	0.55	0.72
		n	19	19	19
		df	17	17	17
	Race/Ethnicity	X^2	95.00	95.00	95.00
		P	0.34	1.00	1.00
		n	19	19	19
		df	85	90	90

Supplemental Table S3. Association of demographic variables with urinary [UA]/[Cr] at baseline, 0-12 hours, and 12-24 hours by oxygen support status. Spearman's ρ tests for continuous variables; Pearson's X^2 tests for race/ethnicity categorical variable; ***P ≤ 0.05**. EGA – Estimated gestational age; CGA – Corrected gestational age; SNAPPE-II – Score for Neonatal Acute Physiology with Perinatal Extension-II.
